# Core procedural skills competencies and the maintenance of procedural skills for medical students: a Delphi study

**DOI:** 10.1186/s12909-022-03323-9

**Published:** 2022-04-09

**Authors:** Patricia Green, Elizabeth J. Edwards, Marion Tower

**Affiliations:** 1 School of Education, Faculty of Humanities and Social Sciences, The University of Queensland, Brisbane, Q 4072 Australia; 2 Faculty of Health Sciences & Medicine, Bond University, Gold Coast, Q 4229 Australia; 3 School of Nursing, Midwifery and Social Work, Faculty of Health and Behavioural Sciences, The University of Queensland, Brisbane, Q 4072 Australia

**Keywords:** Procedural skills, Competencies, Medical students, Maintenance of competency

## Abstract

**Background:**

It is well recognised that medical students need to acquire certain procedural skills during their medical training, however, agreement on the level and acquisition of competency to be achieved in these skills is under debate. Further, the maintenance of competency of procedural skills across medical curricula is often not considered. The purpose of this study was to identify core procedural skills competencies for Australian medical students and to establish the importance of the maintenance of such skills.

**Methods:**

A three-round, online Delphi method was used to identify consensus on competencies of procedural skills for graduating medical students in Australia. In Round 1, an initial structured questionnaire was developed using content identified from the literature. Respondents were thirty-six experts representing medical education and multidisciplinary clinicians involved with medical students undertaking procedural skills, invited to rate their agreement on the inclusion of teaching 74 procedural skills and 11 suggested additional procedures. In Round 2, experts re-appraised the importance of 85 skills and rated the importance of maintenance of competency (i.e., Not at all important to Extremely important). In Round 3, experts rated the level of maintenance of competence (i.e., Observer, Novice, Competent, Proficient) in 46 procedures achieving consensus.

**Results:**

Consensus, defined as > 80% agreement, was established with 46 procedural skills across ten categories: cardiovascular, diagnostic/measurement, gastrointestinal, injections/intravenous, ophthalmic/ENT, respiratory, surgical, trauma, women’s health and urogenital procedures. The procedural skills that established consensus with the highest level of agreement included cardiopulmonary resuscitation, airway management, asepsis and surgical scrub, gown and gloving. The importance for medical students to demonstrate maintenance of competency in all procedural skills was assessed on the 6-point Likert scale with a mean of 5.03.

**Conclusions:**

The findings from the Delphi study provide critical information about procedural skills for the Clinical Practice domain of Australian medical curricula. The inclusion of experts from medical faculty and clinicians enabled opportunities to capture a range of experience independent of medical speciality. These findings demonstrate the importance of maintenance of competency of procedural skills and provides the groundwork for further investigations into monitoring medical students’ skills prior to graduation.

## Background

Training in clinical procedures (i.e., procedural skills) is a fundamental component of medical curricula, but the selection of skills included in medical programs in Australia differs between medical schools. Procedural skills can range from simple tasks, such as taking vital signs, to complex tasks, such as the insertion of an endotracheal tube for intubation. In Australia, the accreditation requirements for medical programs are set by the Australian Medical Council (AMC). The AMC provides guidance for graduate outcome requirements in the Clinical Practice domain and stipulates that medical students are taught *‘a range of common procedural skills’ *[1], p. 9). The current prerequisite is for medical students to develop competency in a range of procedures by graduation to enable them to work safely in complex, dynamic and unpredictable clinical environments [1, 2].

Competency in medicine can be defined as having the knowledge, skills and experience to be able to fulfil the requirements of the role of the medical professional [3]. Universities are tasked with the preparation of medical students by providing training and experiences during their undergraduate medical program [1]. Students are required to achieve competency in a range of skills by graduation to enable them to work safely in complex, dynamic and unpredictable clinical environments [3–5]. Acquiring competency in procedural skills, therefore, is an essential goal of medical education, with the expectation that a graduate should be proficient in basic procedural and clinical skills and able to assume responsibility for safe patient care at entry to the profession. Universities are tasked with preparing medical students for clinical work by providing procedural skills training required to practice as a graduate doctor. However, it is for individual medical programs to decide which procedures are taught and determine the competency level for each skill.

Although there is no national undergraduate medical curriculum in Australia, most medical programs teach procedural skills in the pre-clinical or early stages of the degree in preparation for clinical practice in the latter years of training [1]. The clinical skills curriculum in Australian medical schools is often aligned with the Australian Curriculum Framework for Junior Doctors [6]. This framework outlines the knowledge, skills, and behaviours inclusive of procedural skills required by interns (i.e., after graduation). Given that the suggested skills are for post- rather than pre-graduation, it is possible that they may not align with the level of ability or requirements of medical students to perform these procedures. Nearly a decade ago, the Medical Deans of Australia and New Zealand Inc (MDANZ) comprising the Deans of Australia’s 21 medical schools and the two New Zealand medical schools, reviewed and suggested 58 procedures plus diagnostic and therapeutic skills [7]. There is, however, no published data to support the rigor of the methods used to determine such findings.

Several reviews of clinical curricula have been undertaken internationally. In the UK, medical students are required to demonstrate competency in 23 practical skills and procedures by graduation [8]. In Canada, a review of the medical curriculum was developed with a competency focus designed to identify and describe the abilities required to effectively meet the health care needs of patients, and is being adopted in the undergraduate medical schools to aid graduates to transition into the clinical area more easily [9, 10]. In the US, using a competency-based model, 13 clinical tasks are specified as entrusted professional activities (EPA) i.e., procedural skills that medical graduates should attain [11, 12]. The definition of an EPA is that it is comprised of discrete tasks relevant to the clinical area and competency is acquired in those activities [13]. In Germany, a consensus review identified 289 practical skills [14] to inform the German National Competency-based Catalog of Learning Objectives for Medicine [15]. In Switzerland and Netherlands, similar catalogues have been developed [16, 17]. Nonetheless, the procedural skills curricula developed in other countries may lack applicability in many aspects of an Australian context [6, 18–21].

An increasing number of studies have reported the difficulty in determining when a medical student is competent to undertake a procedural skill, and as such, many new graduates feel inadequately prepared for clinical practice [22, 23]. Recent research reported the lack of competency in procedural skills among graduates and has implicated shortcomings associated with traditional approaches to teaching clinical and procedural skills and the challenges in maintaining competence during medical school [24]. Another study described downstream consequences of such ill-preparedness in clinical and procedural skills for preventable medical errors, infection rates and patient morbidity, all known to be greater in newly graduated doctors [25].

As the paradigm shifts from the long-established, time-based medical education model, grounded in apprentice-type practice with patients in primary care, towards a competency-based model involving mastery learning and competency standards, the importance of acquisition of procedural skills along with the maintenance of competence is becoming increasingly relevant [5, 26]. Competency standards serve several developmental functions (i.e., stages of competency) that promote the minimum requirements for fitness to practice [27]. The standards also direct students towards undertaking responsibility for their own professional development and practice [28]. However, there is a decline in opportunities for attaining clinical experience, partly due to patients not spending as much time in hospital pre- or post-operatively, and partly due to the acuity of inpatients not always being conducive to allowing medical students to practice procedural skills [29]. Further, many procedures once performed by interns and practised by medical students, are now performed by other health practitioners, for example, nurses routinely undertake peripheral intravenous cannulation on wards and suture wounds in the Emergency Department [24] and midwives suture vaginal lacerations and episiotomies in the birthing suite [30].

Sawyer et al. [26] proposed an evidence-based framework for teaching and learning of procedural skills in six steps of ‘*Learn, See, Practice, Prove, Do and Maintain’*. Sawyer et al., highlights the importance of maintenance, arguing that competency in procedural skills ‘degrades’ if practice is not undertaken and/or refreshed. In the undergraduate years of medical curricula, however, individual procedural skills are often only formally assessed in OSCE examinations and not re-assessed. As such, many medical students do not recognise that their level of skills has declined nor realise the importance of maintaining skills. The importance of maintaining competence has long been recognised in other areas of health (e.g., basic and specialised resuscitation skills) and the post-graduate speciality colleges, to ensure skills are maintained and professional development requirements are met [31–33].

Currently, the level of achievement in the learning of procedural skills, focuses on assessment of psychomotor skills and a multidimensional nature of competence, but the translation of the procedure to a range of attributes required for professional practice does not appear to be well considered [34]. The need to review a set of procedural skills competencies that will enable medical students to function more efficiently in the clinical setting, is evident. Importantly, it is likely that once standardised strategies for ensuring student competence in procedural skills is determined, monitoring student outcomes prior to graduation may be required [35–37].

## Method

### Design

The present Australian-based study utilised a three-round modified Delphi technique [38, 39] to explore consensus from a panel of key medical education academics and healthcare clinicians. The purpose of this Delphi study was to identify core procedural skills competencies for Australian medical students and to establish the importance of the maintenance of such skills. The Delphi technique is a well-established hybrid research method that combines both quantitative and qualitative approaches. This method has been used to arrive at group consensus across a range of subject areas, including the field of competencies in clinical education when knowledge of the subject is not well defined or has not been recently addressed [40–42].

Traditionally, the first round of the Delphi technique asks the selected expert panel to consider questions to establish the content required and then to establish consensus [39] whereas the Delphi method used here is considered ‘modified’ as the method used a prepared set of items. Consensus in the Delphi method is developed through successive survey rounds as participants identify their level of agreement and reassess their previous level of agreement [41, 43] or a criterion for stopping is reached [44]. To achieve an acceptable range of consensus, a definition of consensus for this study was set (a priori) at 80% agreement and the number of rounds at three, although there is no uniformity about how to conduct a Delphi, as the number of rounds and the panel size varies [44–47].

Core competency was defined as the essential minimum set of a combination of attributes, such as applied knowledge, skills, and attitudes that enable an individual to perform a set of tasks to an appropriate standard, efficiently, effectively and competently in the profession at the specified level [48]. Core competencies offer a shared language for health professions for defining the expectations of procedural skills competency. In this study, participants were also asked to consider the priorities of the AMC Graduate Outcomes Statement [1] and the definition of competency in making their decisions. Maintenance of competency is defined as the ongoing ability to integrate and apply the knowledge and skills to practise the set of tasks safely in a designated role and setting. Medical professionals are responsible for ensuring they stay up to date on a continuing basis with lifelong learning to meet the requirements of the regulatory body and these standards protect the public as well as advance practice.

### Delphi expert panel

A total of 75 medical academics, clinical educators and clinicians from Australia were invited to participate in this study by email in March 2020. A purposive sampling strategy was used to identify a representative sample of potential experts. Recruitment was by emailing an invitation to experts identified from websites of Australian medical schools, medical students’ placement affiliations (e.g., hospitals), Australian authors of published papers, and snowballing using recommendations by third parties. The email provided the inclusion criteria and information about the Delphi study. Literature has suggested panels with 10 to 50 individuals are appropriate [38], based on similar studies, we anticipated between 25 and 40 experts would agree to take part [49]. To capture the collective opinion of experts in this area, the inclusion criteria for the study were: a medical or health qualification, involved with medical students in clinical and/or educational settings in Australia where procedural skills are undertaken [39]. These settings were selected to access individuals who would have the pre-requisite knowledge and experience with medical students and procedural skills. Equal weight was given to the opinions of each participant. Ethical approval (reference # PG03205) for this study was obtained through the Human Research Ethics Committee of the university. All participants provided informed consent to take part at the beginning of each survey round.

### Data collection

Invited experts accessed survey rounds via a link hosted on the web-based platform Qualtrics, (www.Qualtrics.com). The research questions and information about the Delphi process was provided on the first screen of each survey. Demographic characteristics were collected, and the survey was open for three weeks. Non-responders received a follow-up email reminder at two weeks.

### Pilot of Delphi survey

Each round of the Delphi survey was piloted with a selected group of eight faculty educators and healthcare clinicians who met the Delphi panel inclusion criteria. Given that they were a convenience sample known to the researcher, their responses were not included in the data [39]. Piloting the survey instrument was done to ensure the relevance of the competencies selected for medical students, to identify incongruent and vague statements and suggest corrections and to ensure the usability and acceptability to participants. The pilot panel were not included as participants in the Delphi study.

### Round 1

A comprehensive list of procedural skills competencies was developed from a review of practice standards of existing curricula, guidelines, and frameworks from national and international published studies e.g., MDANZ [7], the GMC UK Practical skills and procedures [8], and a literature search on procedural skills competencies. Key words and phrases included competency, medical students, procedural skills, curricula and Boolean combinations. Databases searched included PubMed, MEDLINE, Web of Science and Scopus. In Round 1, using the description of core competency, experts were asked to consider the question: *‘Should medical students be able to perform these skills?’* Specifically, they were asked to rate a total of 74 procedural skills across ten categories using a three-point scale (*yes, no* or *unsure*) according to whether they considered medical students should achieve a level of competence for each procedure by the end of their medical degree. The categories were: cardiovascular, diagnostic/measurement, gastrointestinal, injections/intravenous, ophthalmic/ENT, respiratory, surgical, trauma, women’s health, and urogenital sections. The option ‘*unsure*’ was included following pilot testing that indicated some participants were unaware of how essential some of the procedures were and preferred to leave the question unanswered. To capture skills that might be considered essential but were not included, the experts were asked to use a free-text box to propose any missing procedures.

### Round 2

In Round 2, experts were provided with findings from Round 1 and invited to clarify and re-rate the relevance of items to determine the level of consensus by answering the question: *Should medical students be able to provide safe treatment to patients through performing these procedures by graduation?* using a six- point Likert scale (*Not at all Important* through to *Extremely Important*) to indicate their level of agreement for inclusion as a requirement by medical students to achieve by graduation. In May 2020, the MDANZ identified a set of core competencies for final year students which aligned with the AMC’s graduate outcomes [50]. Irrespective of consensus, the eighteen procedural skills from the MDANZ guidance statement were included and presented to the experts in Round 2. Round 2 aimed to establish stability with procedural skills that did not achieve > 90% consensus in Round 1 and were re-submitted in Round 2. Skills from Round 1 that achieved > 90% were considered to have reached consensus and it was assumed that agreement was unlikely to alter, therefore they were not represented in Round 2. Additionally, a determination about the importance for students to demonstrate maintenance of competency was assessed on the 6-point Likert scale. The type of maintenance program was also investigated and the timing that would be appropriate for such a program of procedural skills.

### Round 3

In Round 3, experts were invited to re-evaluate the procedures that achieved consensus in the previous rounds for a level of maintenance. As part of a two-part question, experts were invited to establish ‘*If maintenance would be required for the procedure?’,* and *‘If yes, at what level should maintenance be at?’* The scale for the level of maintenance was rated in four levels based on the Dreyfus model of skill acquisition: *Observer* – understands and observes the procedure in the clinical environment, *Novice* – performs the procedure under direct supervision in a simulated environment, *Competent –* performs with supervision nearby in the clinical environment and *Proficient *– performs proficiently under limited supervision in the clinical environment [51]. A *Not Applicable* category was available. A free text box was available for any comments regarding maintenance of competency following each section.

### Data analysis

Descriptive statistics were used to describe experts’ demographic characteristics and group responses to each item in all rounds with frequency statistical data calculated for each item during the rounds. Descriptive statistics (median and interquartile range) were calculated to determine the indicators for selection in the next round and to present quantitative feedback (median and interquartile ranges). Measurement of percentage of agreement, range of ratings (interquartile ratings), mean and median were analysed using IBM SPSS version 26 (IBM, 2016). The appropriate level of consensus is inadequately defined within the literature with measurements ranging from 51 to 100% [52]. Consistent with previous work, in the current study a priori decision to establish consensus was made if 80% or more of experts agreed on an item rating [53, 54].

## Results

### Delphi expert panel

The Delphi rounds were conducted between March 2020 and July 2020. Table 1 displays the characteristics of panel experts at each round. Of the 75 experts contacted, 40 agreed to participate, and 36 completed Round 1. This equated to a response rate of 48%. Those who participated in Round 1 were sent the Round 2 survey, and 33 completed surveys were received, representing a response rate of 92%. Those who participated in Rounds 1 and 2 were sent the Round 3 survey, with a 75% response rate. The consensus opinions, representing an expert group of Australian faculty educators from nine medical schools and healthcare clinicians, with 83% having more than five years’ experience in clinical education.

**Table 1:** Characteristics of panel experts in Round 1, Round 2, and Round 3

	Round 1		Round 2		Round 3	
	*n* = 36	(%)	*n* = 33	(%)	*n* = 27	(%)
**Profession**^a^						
Medicine	14	(38)	18	(54)	17	(63)
Nursing	11	(30)	10	(30)	7	(26)
Medical Education	17	(47)	17	(51)	7	(26)
Other^b^	3	(9)				
Total responses	36	(48)	33	(91)	27	(75)
**Gender**						
Female	27	(75)	24	(72)	22	(81)
Male	9	(25)	9	(28)	5	(19)
**Practice Location**^a^						
ACT	1	(3)				
NSW	4	(11)				
Queensland	28	(78)				
South Australia	2	(6)				
Tasmania	2	(6)				
**Years in clinical education**						
< 5	6	(17)				
6–10	12	(33)				
11–15	6	(17)				
16–20	5	(14)				
> 20	7	(19)				

^a^Some experts responded in more than one category of the same section. For example, experts could work across two professions and across two states (i.e., Northern NSW/Southeast QLD) therefore, total ≠ 100%

^b^These experts were currently working in a medical school: a psychotherapist, a medical researcher (previously registered medical practitioner) and a clinical skills simulator (previously registered nurse)

### Round 1

Round 1 comprised a list of 74 procedural skills. Table 2 shows the consensus of importance of procedural skill competency level rated by the experts. Eleven procedural skills were rated 100% agreement and were not re-submitted in Round 2. Twenty-eight skills achieved 90 – 100% agreement and nine skills scored 80 – 90% and were re-presented in Round 2 for stability of scoring. The specialist categories of ophthalmoscopy, women’s health and urogenital scored the lowest agreement (see Table 2 for the procedural skills not re-submitted in Round 2). Following analysis of experts’ suggestions and reconciliation with the MDANZ guidance statement [50] 21 skills were added, and one removed as it was not a procedural skill according to the definition of a procedural skill (prescription of intravenous fluids). Additionally, six skills were combined, namely, male and female catheterisation were combined into one skill, perform and interpret an ECG were combined into one skill, and maintaining an airway and basic airway management was combined.

**Table 2:** Consensus of importance of procedural skill competency by graduation presented in Round 1, and level of agreement from Round 2

**No**	**Procedural Skill**	**Round 1** **importance**^**a**^ *n* = 36	**Round 1** **% agreement**	**Round 2** ***Mdn* importance**^**b**^** (IQR)** *n* = 33	**Round 2** ***M* importance**^**c**^** (SD)**	**Round 2** **% agreement**
**Cardiopulmonary**						
1	Advanced life support	N	48	3 (3–5)	3.44 (1.26)	47
2^c^	Basic life support	Y	100	-	-	
3	Cardiopulmonary resuscitation	Y	100	-	-	
4^c^	Electrocardiogram	Y	97	-	-	
5^c^	ECG – interpretation	Y	97	-	-	
6^c^	Defibrillation	Y	88	5.5 (4–6)	5.03 (1.19)	84
7	Basic First Aid	Y	97	-	-	
**Diagnostic/measurement**						
8^c^	Arterial blood gas	Y	65	4 (3.75–5)	4.21 (1.32)	70
9^c^	Blood culture (peripheral)	Y	88	5 (4–6)	5.09 (1.06)	85
10	Blood glucose level (BGL)	Y	94	-	-	
11	Body Mass Index – calculation & interpretation	Y	91	-	-	
12	Doppler arterial assessment	N	45	4 (3–4)	3.62 (1.18)	60
13	Observations – TPR & BP	Y	100	-	-	
14	Pulse oximetry	Y	91	-	-	
15^c^	Urinalysis	Y	97	-	-	
75	Ankle Brachial Index	-	-	4 (2–5)	3.56 (1.40)	59
**Gastrointestinal**						
16	Digital rectal examination	Y	88	5 (4–6)	4.91 (1.11)	82
17	Nasogastric insertion	Y	82	5 (4–6)	5.00 (0.85)	83
18	Proctoscopy	N	5	2 (1–4)	2.59 (1.37)	43
19	Sigmoidoscopy	N	0	2 (1–3)	2.12 (1.20)	35
**Injections/Intravenous**						
20	Central line insertion	N	14	2 (2–4)	2.71 (1.49)	45
21	Central venous pressure measurement	N	34	3 (2–4)	3.21 (1.45)	53
22	Digital block – toe or finger	N	48	4 (3–5)	4.18 (1.24)	35
23	Local anaesthetic injection	Y	91	-	-	
24^c^	Intramuscular injection	Y	100	-	-	
25^c^	Peripheral intravenous cannulation& infusion	Y	97	-	-	
26^c^	Subcutaneous injection	Y	97	-	-	
27^c^	Venepuncture	Y	94	-	-	
28	Blood transfusion procedure	N	57	5 (4–6)	4.68 (1.43)	78
29	Ultrasound guided venous access	N	45	4 (3–5)	3.88 (1.27)	65
30	Prescription of IV fluids	Y	91	-	-	
76^d^	PICC line	-		2 (2–4)	2.71 (1.32)	
77^d^	Portacath insertion	-		4 (2.75–5)	3.71 (1.55)	
**Ophthalmic/ENT**						
31	Foreign body removal – ear or nose	N	68	5 (4–5)	4.58 (1.09)	75
32	Eyelid eversion	N	68	5 (4–6)	4.79 (1.11)	80
33	Ophthalmoscopy	N	74	5 (4–5)	4.58 (1.06)	76
34	Otoscopy	Y	88	5 (5–6)	5.18 (0.85)	86
35	Posterior nasal pack insertion for epistaxis	N	54	4 (3–5)	4 (1.15)	66
36	Slit lamp use	N	51	4 (3–5)	4.15 (1.15)	69
**Respiratory**						
37^c^	Basic airway management	Y	100	-	-	
38	Use of equipment e.g., asthma inhalers	Y	100	-	-	
39^c^	Laryngeal mask airway	Y	80	5 (4–6)	4.61 (1.62)	77
40	Peak flow function testing	Y	94	-	-	
41	Pleural biopsy	N	5	2 (1–3)	2.09 (1.47)	35
42	Spirometry	Y	82	5 (4–6)	4.61 (1.62)	77
43	Use of equipment e.g., oxygen delivery devices	Y	97	-	-	
44^c^	Guedel airway insertion	Y	85	5 (4–5)	4.64 (1.14)	77
45	Bag & mask ventilation	Y	97	-	-	
46	Endotracheal placement	N	31	3 (2–5)	3.45 (1.56)	57
78^d^	Nasopharyngeal airway		-	5 (4–6)	4.7 (1.38)	78
79^d^	Supraglottic airway		-	4 (3–5)	4.06 (1.52)	68
80^d^	Maintaining an artificial airway e.g., suctioning		-	5 (4–6)	5 (1.30)	83
**Surgical**						
47^c^	Asepsis – wound care and management	Y	100	-	-	
48	Aspiration	N	60	4 (3.5–5)	4.06 (1.14)	68
49	Infection control – PPE	Y	100	6 (6–6)	6 (.00)	100
50	Skin biopsy – shave, punch & excisional	N	54	4 (3–5)	4 1.44)	66
51^c^	Suture insertion	Y	100	-	-	
52	Suture removal	Y	97	-	-	
53	Scrub, gown & glove	Y	100	-	-	
**Trauma**						
54	Chest drain insertion	N	22	3 (2–4)	3.27 (1.35)	54
55	Glasgow coma scale	Y	100	-	-	
56	Joint aspiration	N	20	3 (2–4)	3.12 (1.11)	52
57	Laceration repair	Y	85	5 (5–6)	5.27 (.80)	88
58	Lumbar puncture	N	34	3 (2–5)	3.26 (1.45)	54
59	Plaster cast/splint limb immobilisation	Y	91	-	-	
60	Removal of foreign body e.g., fishhook or glass	N	74	5 (4–5)	4.67 (1.02)	78
81^d^	Intraosseous insertion		-	4 (2–5)	3.88 (1.64)	65
82^d^	Staples/glue for wound closure		-	6 (5–6)	5.27 (.94)	88
83^d^	Dislocation		-	4 (4–5)	4.42 (1.09)	74
**Urogenital**						
61	Cystoscopy	N	5	1 (1–3)	2 (1.32)	33
62	Paraphimosis reduction	N	37	3 (2–5)	3.27 (1.61)	54
63	Suprapubic catheterisation	N	25	3 (2.5 -4)	3.24 (1.32)	54
64^c^	Urethral catheterisation – female	Y	97	5.45 (5–6)	6 (1.03)	100
65^c^	Urethral catheterisation – male	Y	97	5.45 (5–6)	6 (1.03)	100
84^d^	Bladder scan		-	5 (3–6)	4.3 (1.81)	72
**Women’s Health**						
66	Abdominal palpation in pregnancy	Y	91	-	-	
67	Abdominal ultrasound	N	51	4 (2–5)	3.45 (1.52)	57
68	Cardiotocography (CTG)	N	60	4 (3–5)	4.15 (1.33)	69
69	Pregnancy urine testing	Y	100	-	-	
70	Vaginal/pelvic examination	Y	94	-	-	
71	Vaginal birth	N	65	5 (4–6)	4.76 (1.15)	79
72	Speculum & pelvic examination	Y	88	5 (5–6)	5.18 (.92)	86
73	Cervical screening test	Y	85	5 (4.5 – 6)	5.06 (.90)	84
74	Breast examination	Y	94	-	-	
85^d^	Endocervical screen		-	5 (4.5 – 6)	5.06 (.90)	84

^a^Y = Yes, N = No, U = Unsure

^b^1 = Not at all important, 2 = Low importance, 3 = Slightly important, 4 = Moderately important, 5 = Very important, 6 = Extremely important

^c^Included in MDANZ Guidance Statement 2020

^d^Included due to expert recommendations from Round 1

Mean of 4.8 = 80%

### Round 2

In Round 2, 54 procedures were considered by the experts who identified 25 procedures as being *very or extremely important* for medical students’ competency (see Table 2). Fourteen procedures did not establish consensus in importance, 12 were identified as having *slight or low importance* and one (cystoscopy) was ranked as *not at all important*. One procedure in the newly published MDANZ guidance [50], arterial blood gas, was included in Rounds 2 and 3 although not considered important in Round 1 but was deemed to require consideration. No further procedures were recommended after the first round. The importance of medical students demonstrating maintenance of competency of all procedures was rated on the 6-point Likert scale with a mean of 5.03. Some form of maintenance program was identified by 55% of the experts, with the majority creating a pre-intern program of between 3–5 procedures. The question about the intervals of a maintenance programme of procedural skills to be assessed/reviewed was variably reported as between every 6 months to annually and prior to graduation as shown in Table 3.

**Table 3:** Importance of maintaining competency in procedural skills and interval to maintain competency

Importance of maintaining competency	*M*	Median	Median response in importance scale^a^	IQR
*N* = 33	5.03	5	Very important	5,6
**Interval of maintaining competency at level of proficient**	**Every 6 months**	**Every year**	**Immediately prior to graduation**	**All of the intervals**
Frequency (%)	3 (11)	13 (48)	18 (66)	4 (14)

1 = Not at all important, 2 = Low importance, 3 = Slightly important, 4 = Moderately important, 5 = Very important, 6 = Extremely important

### Round 3

An individual summary of the Round 2 median scores for each procedural competency plus the median group results were provided separately to each participant prior to the Round 3 survey. Round 3 explored the level of importance of maintenance for the final set of core procedural skill and the level of maintenance (i.e., observer, novice, competent, proficient). Table 4 shows that 41 procedural skills were considered to require maintenance at a proficient or competent level, 14 with 100% agreement. Four procedures achieved between 70—77% agreement but did not establish the threshold for consensus in maintenance of competency. The levels of maintenance showed variability in the selections.

**Table 4:** Level of importance and agreement of maintenance of procedural skill presented in Round 2, and level of maintenance from Round 3 listed in order of importance

No	Procedural Skill	R2 Mdn maintenance^a^ (IQR)	R2 *M* maintenance^a^ (SD)	R2 % agreement	R3 maintenance^b^	R3 % agreement	R3 level^c^ (%)
2	Basic life support	-	-	-	Y	100	O (O), N (7), C (30), **P (63)**
3	Cardiopulmonary resuscitation	-	-	-	Y	100	O (0), N (4), **C (55)**, P (41)
13	Observations – TPR & BP	-	-	-	Y	100	O (4), N (0), C (22), **P (74)**
14	Pulse oximetry	-	-	-	Y	100	O (4), N (0), C (22), **P (74)**
23	Local anaesthetic injection	-	-	-	Y	100	O (0), N (26), **C (41)**, P (33)
24	Intramuscular injection	-	-	-	Y	100	O (0), N (11), C (41), **P (48)**
26	Subcutaneous injection	-	-	-	Y	100	O (7), N (7), C (41), **P (45)**
43	Use of equipment e.g., oxygen delivery devices	-	-	-	Y	100	O (0), N (7), **C (56)**, P (37)
45	Bag & mask ventilation	-	-	-	Y	100	O (0), N (15), **C (45)**, P (40)
47	Asepsis – wound care & management	-	-	-	Y	100	O (0), N (7), C (45), **P (48)**
49	Infection control—PPE	6 (6–6)	6 (.00)	100	Y	100	O (0), N (4), C (44), **P (52)**
51	Suture insertion	-	-	-	Y	100	O (0), N (7), **C (55)**, P (37)
53	Scrub, gown & glove	-	-	-	Y	100	O (0), N (4), C (41), **P (55)**
55	Glasgow coma scale	-	-	-	Y	100	O (0), N (7), C (45), **P (48)**
4	Electrocardiogram (ECG)	-	-	-	Y	96.3	O (0), N (7), **C (52),** P (37) NA (4)
6	Defibrillation	5.5 (4–6)	5.03 (1.19)		Y	96.3	U (4), O (4), N (18), **C (48),** P (26)
7	Basic First Aid	-	-	-	Y	96.3	O (0), N (0), C (37), **P (60),** NA (3)
10	Blood glucose level (BGL)	-	-	-	Y	96.3	O (4), N (0), C (37), **P (59)**
27	Venepuncture	-	-	-	Y	96.3	O (0), N (15), C (26), **P (55),** NA (4)
37	Basic airway management	-	-	-	Y	96.3	O (0), N (11), C (37), **P (48),** NA (4)
38	Use of equipment e.g., asthma inhalers	-	-	-	Y	96.3	O (0), N (4), **C (48),** P (44), NA (4)
52	Suture removal	-	-	-	Y	96.3	O (0), N (11), C (26), **P (59),** NA (4)
64	Urethral catheterisation – female	5.45 (5–6)	6 (1.03)	-	Y	96.3	O (0), N (22), **C (52),** P (26)
74	Breast examination	-	-	-	Y	96.3	O (0), N (22), **C (48),** P (26), NA (4)
25	Peripheral intravenous cannulation & infusion	-	-	-	Y	92.6	O (4), N (15), C (33), **P (40),** NA (7)
34	Otoscopy	5 (5–6)	5.18 (.84)	-	Y	92.6	O (4), N (11), **C (59),** P (26)
57	Laceration repair	5 (5–6)	5.27 (.80)	-	Y	92.6	O (0), N (30), **C (52),** P (15), NA (3)
65	Urethral catheterisation – male	5.45 (5–6)	6 (1.03)	-	Y	92.6	O (0), N (22), **C (56),** P (18), NA (4)
9	Blood culture (peripheral)	5 (4–6)	5.09 (1.06)		Y	88.9	O (4), N (18), C (33), **P (37),** NA (8)
15	Urinalysis	-	-	-	Y	88.9	O (0), N (0), C (26), **P (63),** NA (11)
39	Laryngeal mask airway	5 (4–6)	4.61 (1.62)		Y	88.9	O (7), N (26), **C (48),** P (15), NA (4)
44	Guedel airway insertion	5 (4–5)	4.64 (1.14)		Y	88.9	O (4), N (7), **C (41), P (41),** NA (7)
82	Staples/glue for wound closure	6 (5–6)	5.27 (.94)		Y	88.9	O (4), N (22), **C (40),** P (26), NA (8)
66	Abdominal palpation in pregnancy	-	-		Y	88.9	O (0), N (18), **C (52),** P (26), NA (4)
70	Vaginal/pelvic examination	-	-		Y	88.9	O (4), N (33), **C (44),** P (15), NA (4)
71	Vaginal birth	5 (4–6)	4.76 (1.15)		Y	88.9	O (11), **N (59),** C (30), P (0)
11	Body Mass Index – calculation & interpretation	-	-		Y	85.2	O (0), N (7), C (11), **P (66),** NA (14)
16	Digital rectal examination	5 (4–6)	4.91 (1.11)		Y	85.2	O (0), N (22), **C (40),** P (30), NA (8)
69	Pregnancy urine testing	-	-		Y	85.2	O (4), N (4), C (30), **P (48),** NA (14)
73	Cervical screening test	5 (4.5 – 6)	5.06 (.90)		Y	85.2	O (0), N (33), **C (44),** P (11)
8	Arterial blood gas	4 (3.75–5)	4.21 (1.32)		Y	81.5	O (15), N (22), **C (37),** P (19), NA (7)
17	Nasogastric insertion & safe placement	5 (4–6)	5.00 (1.43)		Y	81.5	O (4), N (26), **C (44),** P (15), NA (11)
40	Peak flow function testing	-	-		N	77.8	O (0), N (15), **C (44),** P (30), NA (11)
42	Spirometry	5 (4–6)	4.61 (1.62)		N	74.1	O (0), N (30), **C (33),** P (22), NA (15)
59	Plaster cast/splint limb immobilisation	-	-		N	74.1	O (4), **N (44),** C (33), P (7), NA (12)
32	Eyelid eversion	5 (4–6)	4.79 (1.11)		N	70.4	O (8), N (22), **C (33),** P (22), NA (15)

^a^1 = Not at all important, 2 = Low importance, 3 = Slightly important, 4 = Moderately important, 5 = Very important, 6 = Extremely important

^b^Y = Yes, N = No, U = Unsure

^c^O = Observer, N = Novice, C = Competent, P = Proficient, U = Unsure, NA = Not applicable

## Discussion

The purpose of this Delphi study was to identify core procedural competencies for Australian medical students and to establish the level of importance of maintenance of such skills. To our knowledge, this is the first study to explore and achieve consensus on the requirement to maintain competency in identified procedural skills and to what level, in the Australian context. We deployed a three-round Delphi technique resulting in a final list of 46 procedural skills representing the consensus opinion of an expert group of Australian faculty educators and healthcare clinicians. Importantly, experts agreed on the importance of competence, acknowledged that skills decay and that continued practice is required to maintain competency.

Our findings provide critical information about the essential procedural skills integral to the Clinical Practice domain of Australian medical curricula. Importantly, they reveal agreement to ensure graduates are able to: *select and perform safely a range of common procedural skills *as required by the AMC [1]. Reconciling our findings with other guidelines/catalogues reveals general agreement. All procedures on the MDANZ guidance statement of clinical practice [50] achieved consensus in our study (i.e., they are within the listed 46 skills). We also established consensus for other skills e.g., vaginal birth, otoscopy, breast examination, and insertion of a Guedel airway. Such differences may reflect changes in the roles of medical students and interns since the Australian Junior Doctor Framework was published in 2009 [6]. There was agreement that procedures such as intravenous drug administration, diagnosis of pregnancy, corneal and foreign body removal and skin lesion excision should remain at an intern role level [6].

Our findings concur with all recommended practical skills and procedures from the UK’s GMC revised 2019 list [8] and the majority of the clinical-practical skills in the German National competency-based learning objective catalog medicine [15] and the Dutch nested EPAs [55]. Surprisingly, the GMC graduate outcomes list of practical skills does not feature basic life support or cardiorespiratory procedures although most UK medical schools do provide some form of compulsory life support training [56]. The number of practical skills and procedures that a UK graduate must know and be able to do has reduced from 32 practical procedures in 2014 (to 23 in 2019) [8].

In Australia, upon successful completion of an accredited medical program, graduates complete a mandatory internship year. Interns may demonstrate procedural lapses and/or practice areas of risk which must be remediated prior to full registration [57]. There are, however, no Australian national requirements to demonstrate procedural competency for registration. By comparison, in other countries regulatory bodies provide a clear catalogue of practical skills and procedures accompanied by minimum levels of proficiency for safe practice [58]. Significantly, our findings highlight that experts view maintenance of competency as essential for professional growth and confidence, and for the safety of patients [22]. We hope the findings from the present study become the catalyst for further research exploring factors that benefit students understanding of maintenance of procedural skills.

The present study has some limitations. Thirty-six skills did not reach consensus from our expert panel. For example, lumbar puncture, proctoscopy and sigmoidoscopy, central line insertion, endotracheal placement, fell short of reaching 80% consensus, which does concur with previous studies [24, 59]. There are several reasons why this might be the case. We were specifically interested in preparation of students for learning on a continuum to achieve and maintain competency in the Clinical Practice domain of Australian medical curricula. Barr and Graffeo’s [24] study was conducted in US, and Monrouxe et al.’s. [59] study was conducted in UK; therefore, it may be due to differences across countries. Given that many hospitals have staff to perform dedicated services (e.g., intravenous cannulation and PICC line insertion), it may be that some specialist procedures were not perceived as a competency requirement for medical students. We drew on a national network of academics and clinicians and gathered views from a range of disciplines, however, it is possible that the size and composition of the expert panel may not have been representative of all medical schools and states. Further, our panel comprised of faculty and practicing health care clinicians which may have contributed to unknown expectations of medical students’ role in specialty areas such as ophthalmology, urology and women’s health and a disproportionate involvement in conveying practical skills in cardiovascular, respiratory and trauma areas.

Medical educators engaged with interest in our consensus study with a high retention of participants in the three rounds, and importantly there exists agreement about the core features of procedural skills training (i.e., skills being taught, level of competency, importance of maintenance). It is not known if this lack of preparedness during internship is due to a decline in practical skills teaching or the maintenance of competency in the curricula of medical students [60]. Our findings highlighted areas where there is less certainty in the requirements for medical students’ competency related to procedural skills, potentially requiring further exploration to examine this. Furthermore, our findings support Sawyer’s evidence-based framework suggesting the importance of maintaining skill levels [26]. An area of future research is to explore how students are currently maintaining their competence with semi-structured interviews. We are currently undertaking this work.

## Conclusions

The present study used a modified Delphi method to establish consensus of 46 procedural skills to underpin the core competencies required for Australian medical students by graduation. Our findings support the importance teaching and maintenance of competency in these procedures within the pre-clinical years of medical curricula and beyond, aligning with the change to an outcomes model of competency-based medical education. Our findings highlight the importance of maintenance to alleviate decay in procedural skills reported in the literature. We suggest that valuing the importance of maintaining skills competency improves patient care and demonstrates attributes of twenty-first century sustainable medical professionals who work as safe, functional practitioners.

## Data Availability

The datasets used and analysed during the current study are available from the corresponding author on reasonable request.
